# Personalized glucose prediction using *in situ* data only

**DOI:** 10.3389/fnut.2025.1539118

**Published:** 2025-06-09

**Authors:** Rohan Singh, Marouane Toumi, Marcel Salathé

**Affiliations:** Digital Epidemiology Lab, School of Life Sciences, School of Computer and Communication Sciences, EPFL, Lausanne, Switzerland

**Keywords:** personalized nutrition, real-world data, real-world evidence, digital cohort, gut microbiome

## Abstract

The worldwide rise in blood glucose levels is a major health concern, as various metabolic diseases become increasingly common. Diet, a modifiable health behaviour, is a primary target for the preventive management of glucose levels. Recent studies have shown that blood glucose responses after meals (post-prandial glucose responses, PPGR) can vary greatly among individuals, even with identical food consumption, and demonstrated accurate PPGR prediction using various features like microbiome data and blood parameters. Our study addresses whether accurate PPGR prediction can be achieved with a limited and easily obtainable set of data collected in real-world, everyday settings. Here, we show that a machine learning algorithm with such real-world data (RWD) collected from a digital cohort with over 1,000 participants can achieve high accuracy in PPGR prediction. Interestingly, we find that the best PPGR prediction model only required glycemic and temporally resolved diet data. This ability to predict PPGR accurately without the need for biological lab analysis offers a path toward highly scalable personalized nutrition and glucose management strategies.

## Introduction

Inadequately controlled blood glucose can lead to severe health issues, including cardiovascular diseases, kidney complications, and nerve damage. Diabetes, defined as a chronic metabolic disorder with high blood glucose levels, represents a significant global health concern. Alarmingly, the already high prevalence of diabetes is on the rise. According to the International Diabetes Federation, around 537 million adults were living with diabetes in International Diabetes Federation (1), a figure expected to reach 783 million by 2045.^1^ The economic impact of diabetes, encompassing both the cost of care and related productivity losses, presents substantial challenges to healthcare systems worldwide.

Continuous Glucose Monitoring (CGM) systems have revolutionized the monitoring of glycemic responses, offering personalized, real-time insights into blood glucose fluctuations. These devices are particularly valuable in research for understanding individual responses to dietary intake, since diet plays a crucial role in managing and potentially preventing diabetes, especially type 2 diabetes (T2D). CGM data have been used in several landmark studies to elucidate the intricate relationship between nutrition and PPGR. For instance, Zeevi et al., in their seminal study (2015) demonstrated that glycemic responses to identical meals vary significantly among individuals, underscoring the need for personalized dietary recommendations.

Aspects of glucose metabolism, like postprandial glucose responses (PPGR) and the glycemic index (GI) of foods, are often assessed using the incremental area under the curve (iAUC) of blood glucose levels over a 2-h window following food consumption. Macronutrients are primary influencers of PPGR, with carbohydrates being the most predominant, affecting mealtime insulin doses in diabetic individuals. Additionally, Gentilcore et al. (2) found that ingesting fat prior to a carbohydrate meal markedly slows gastric emptying and attenuates postprandial rises in glucose. Another study examined the effects of both protein and fat on glycemic responses, noting that protein and fat independently reduce the glycemic response elicited by oral glucose. Interestingly, gram-for-gram, protein had a 2–3 times larger effect on reducing glycemic responses than fat (3). This finding indicates the potential for dietary protein to significantly modulate postprandial glycemic spikes, albeit the effectiveness might vary with individual physiological factors such as waist circumference and fasting plasma insulin levels.

Recent studies using continuous glucose monitoring (CGM) and meal logging to predict PPGR—typically using decision tree machine learning models—supported the prospects of personalized nutrition and diabetes management (4–7, 8). These studies harness an array of features from a myriad of data sources such as dietary nutritional composition, physiological characteristics of participants (i.e., age, BMI etc.), blood and serological information, or physical activities and microbiome data, to forecast individual glycemic responses with high accuracy. Notably, these studies emphasized inter-individual variability in postprandial responses to identical meals (5, 8).

Zeevi et al. (4) demonstrated that personalized dietary recommendations based on machine learning model predictions could significantly lower PPGR levels. This approach was substantiated by Berry et al. (5), whose predictive framework included comprehensive inputs like including baseline characteristics and genetic factors. Their findings also indicated that similar meals consumed at different times of the day elicited different glycemic responses, emphasizing the role of circadian rhythms and timing in metabolic regulation. Additionally, Mendes-Soares et al. (6) utilized a variety of dietary, microbiome, and physiological parameters to capture the nuanced nature of glycemic response. Meanwhile, Tily et al. (7) research incorporated individual variations in PPGR to the same foods, stressing the importance of metatranscriptomics features in improving the model performance. Finally, the Sondertoft et al. (8) study, involving standardized meals, emphasized the importance of microbiome and blood clinical features boosting their model’s performance.

While these studies have provided valuable insights, questions remain about the minimal set of data required for accurate PPGR prediction in real-world settings. Our study addresses this gap by leveraging a large digital nutritional cohort, “Food & You” (9), comprising over a thousand participants. We wanted to understand whether accurate PPGR prediction could be achieved using a limited set of easily obtainable data collected by participants in their daily lives, spanning nutritional, glycemic, physiological, anthropometric, and microbiota data. This real-world data enabled us to evaluate the potential for improving prediction accuracy while maintaining scalability, especially when considering normal, non-standardized meals. Overall, we find that the most accurate PPGR prediction model only requires glycemic and dietary information. This finding points to scalability of our approach compared to those that require biological samples and subsequent lab analyses.

## Materials and methods

### Participants and data collection

**FIGURE 1:**
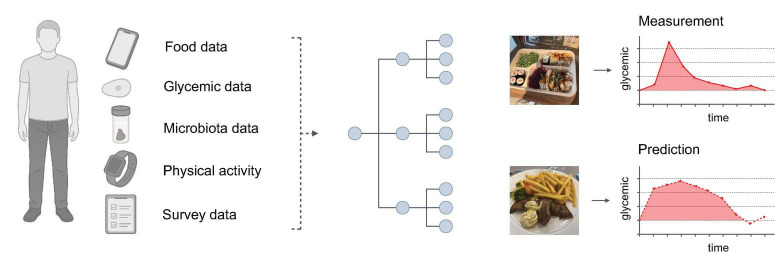
Study setup. **Left:** The Food and You digital cohort collected data on diet, glycemia, microbiota, physical activity, and other factors. **Middle:** These data are used to train a machine learning model (gradient-boosted trees) for PPGR prediction. **Right:** Each meal has a corresponding PPGR value measured as incremental area under the curve (iAUC, see Materials and methods). The model tries to predict the iAUC of a given meal - iAUC predictions and iAUC measurements are then compared to assess model performance.

The “Food and You” study, conducted in Switzerland, is a digital nutrition cohort where participants, over 2–4 weeks, use mobile applications and sensors to monitor their food intake, physical activity, and glycemia (9). All interactions and data collection are digitally facilitated, leveraging the potential of fully remote, “*in situ*” studies in epidemiological research. Participants utilized the MyFoodRepo app for real-time food tracking, continuous glucose monitors to record blood sugar levels, and either activity trackers or surveys to track physical activity and sleep. The study also involved the collection of a one-time stool sample for gut microbiota. Overall, the study collected the following individual features (Figure 1).

1. *Nutrition*: The daily detailed food consumption data of all participants was collected over a minimum of 14 days under real-world conditions, avoiding the constraints of hospital or center visits. This was accomplished using an AI-assisted mobile application, MyFoodRepo, which enabled participants to record their meals in real time through various methods: Photographing their meals, scanning barcodes, or manual entry. When meals were photographed, the app employed a food recognition AI model to identify the contents of the meals and provide corresponding nutritional information. Each time stamped food data entry was verified by a human annotator.
2. *Glucose measurements*: Flash Glucose Monitor Freestyle Libre (Abbott Diabetes Care) were worn by all participants at the start of the study which measured blood glucose levels every 15 min up to 14 days.
3. *Gut microbiome*: The “Food and You” study collected nearly 1,000 stool samples from participants, of both Swiss and foreign origin. These samples were then dispatched to a third-party company (MicroSynth) for 16S sequencing. QIIME2 (10) was used to process the raw sequencing to obtain relative abundances of taxa at different taxonomic levels (see Microbiome Features Processing section for detailed information).
4. *Physical activity and sleep data*: Study participants’ physical activity and sleep data were collected either objectively through Apple Health, Google Fit, or smartwatches, or subjectively *via* self-reports on the study website. The gathered data included daily step count, calories burned, bedtime, wake-up time, and specifics of physical activity.
5. *Personal attributes*: We also collected relevant personal information such as age, weight, ethnicity, income, household description, past diseases, antibiotics used, stress levels, and many others. The variables are provided in Supplementary Table 1.

Participants in the “Food & You” study were required to consume standardized breakfasts from 2 to 7 days of the first week (and days 16–21 for Cohort C participants). The standardized breakfasts varied by day and dietary restrictions: days 2–3 included white bread (or gluten-free for those with restrictions), days 4–5 included white bread with butter or dark chocolate, and days 6–7 consisted of 50 g glucose drinks for all participants. Each standardized meal contained approximately 50 g of carbohydrates. Participants were instructed to avoid altering the standardized meals and to refrain from eating or physical activity for 2 h after consumption.

### Food feature processing

Because our data analysis pipeline required that meals have at least 12 h of glucose data before and after meal intake, dietary data was trimmed to include meals in the time window *t*_*start*_ to *t*_*end*_, where *t*_*start*_ is 12 h after the first glucose measurement, and *t*_*end*_ is 12 h before the last glucose measurement. If multiple meals were consumed by a participant within a 30-min interval, they were merged and their nutritional compositions were summed.

Two different feature sets for nutritional data were constructed, temporal and compositional. Temporal features include features such as meal timing, time since last meal, carbohydrates consumed in the past 6 h, etc. Computation of past temporal nutritional features was obtained by aggregating nutrient information at various time intervals (1, 2, 3, 6, 12 h) prior to each meal. Meal timings were assigned as breakfast (for meals consumed before 10:00), lunch (between 10:00 and 16:00), or dinner (after 16:00).

Non-temporal food features included macro- and micronutrient features, such as carbohydrate content, eaten quantity, fat-carb ratio, etc. Additionally, meals were classified into related food classes (namely *dairy_products_meat_fish_eggs_tofu, vegetables_fruits, sweets_salty_snacks_alcohol, non-_alcoholic_beverages, grains_potatoes_pulses, oils_fats_nuts*). Amounts (in grams) of a meal in these food classes were also taken as non-temporal features. For fat-to-carb ratios and protein-to-carb ratios, meals with zero carbohydrates were assigned zero for these features.

### Glucose data synchronization and processing

Incremental area under the curve (iAUC) was calculated by measuring the area under the curve in a 2 h window over a baseline glucose value for every logged meal (11). Potential challenges, such as time gaps between glucose readings and meal times, were systematically addressed during the computation of postprandial glucose responses, since extracting baseline glucose value for any logged meal to compute iAUC is critical for accurate assessment of PPGR. If glucose measurements were within 30 min of time difference, then the mean value between the measurements was used to fill the gap. Meals were excluded from analysis if time gaps exceeding 30 min were observed for any of the glucose feature calculations. Due to 15 min time intervals between glucose measurements using Abbott Freestyle Libre glucose sensors, and the possibility that people may sometimes have had a small delay in their meal loggings, we implemented a minima-based approach to identify the closest local minima as glucose baseline relative to each mealtime, and use that time point to calculate the 2 h iAUC. We investigated different search time windows into the past around standardized meals (Supplementary Figure 1). We observed that 30 min yielded a much larger increase in the iAUC when compared to the iAUC derived from a 15 min time window, while the difference of the median iAUC values for 45 min and 60 min did not differ significantly from the 30 min window. Hence, we chose a 30 min time window to search for the closest local minima glucose value for the baseline to calculate iAUC.

Glucose variables were computed by analyzing the temporal relationship between meal start times and corresponding glucose values for each participant. This yields the glucose trend and incremental area under the curve (iAUC) trends over different time intervals (1, 2, 4 h) prior to a logged meal, enriching the dataset with detailed glycemic information. Only those observations with non-null values in the glucose trends and previous hour iAUCs were retained.

### Microbiome features processing

Each participant provided one stool sample anytime during the tracking days phase of the Food & You study. Collected samples were shipped to Microsynth AG (Balgach, Switzerland) in batches of 100–192 samples for sequencing. V4 region of the bacterial 16S rRNA gene was sequenced *via* creation of two-step Nextera PCR libraries using the primer pair 515F (NNNNNGTGYCAGCMGCCGCGGTAA) and 806R (NNNNNGGACTACNVGGGTWTCTAAT). After obtaining the sequencing reads from Microsynth, we used QIIME 2 (version 2024.2) for microbiome preprocessing and feature generation. The preprocessing was done by importing demultiplexed single-end sequencing reads, adhering to the Casava 1.8 format, into the QIIME 2 environment. Deblur (version 2024.2) (12) was utilized to denoise sequences and construct amplicon sequence variants (ASVs). We applied a left-trim of 24 bases to remove the primer, and a trim length to constrain to the ASVs to the first 150 bp. These 150 bp ASVs were searched against Greengenes2 (version 2022.10) (13) using q2-greengenes2 (version 2024.1) to obtain a phylogenetic taxonomy. Rarefaction at a sampling depth of 15,183 was performed to normalize library depth. This sampling depth resulted in 8 samples being excluded to low sampling depth while retaining 992 samples for downstream analysis. We additionally applied PICRUST2 (version 2.5.2) (14) for functional and pathway prediction of the microbial sequences, which yielded predicted enzyme commission (EC) numbers and Kyoto Encyclopedia of Genes and Genomes (KEGG) Orthology (KO) groups.

Unweighted UniFrac (15) was computed with q2-diversity (version 2024.2), followed by computing the first 10 principal coordinates. Additionally, we incorporated alpha diversities through q2-diversity, namely, Faiths phylogenetic distance (16), Pielou’s evenness, observed features, and Shannon diversity (17). We also considered use of the microbial ASV table directly and as PCA features (30 components), however, since they did not improve the model performance, we opted to only show results using principal coordinates from unweighted UniFrac distances.

### Sleep and physical activity features processing

For sleep and physical activity data, we extracted two types of temporal features: (1) the duration of each event (time elapsed from start to end of sleep/activity), and (2) the time intervals between the event boundaries and meal logging times (specifically, how much time had passed from the start of the activity to the meal logging, and from the end of the activity to the meal logging). Because providing sleep and physical data was optional in the Food & You study, they had lower completion rates compared to food and glucose data. Subjective activity data (i.e., data collected *via* daily questionnaires) had the largest coverage among participants and were therefore used to build the features.

### Past cumulative glucose metrics

With the help of the “iglu” package (version 3.4) in R (18), different glucose metrics, such as ADRR, LBGI, HBGI, COGI, CONGA etc., were computed for each participant (Supplementary Table 1). This was done cumulatively across all data collection days of the participant, i.e., for a given day, glucose data for all previous days were used to compute the relevant glucose metrics. Hence, such glucose metrics data could only be generated from the 2nd day onward for all participants.

### Model training parameters

The XGBoost regression model [version 2.0, (19)] was trained using the GroupShuffleSplit cross-validation method with parameters set as follows: test size of 0.2, 5 splits, and a random seed of 42. A grid search for hyperparameter tuning was conducted for each model combination, searching for the optimal number of estimators within the range of 1,000–4,000, learning rate between 0.001 and 0.01, maximum depth among 6, 7, 8, subsample ratio from 0.2 to 0.9, and column subsampling rates of 0.3, 0.6, and 0.9. The model’s performance was assessed through Pearson correlation R comparing predicted iAUC with actual iAUC values. Different hyperparameters for different model combinations are provided in Supplementary Table 2.

### Variance explanation

A linear regression model was fitted to assess the impact of selected factors on a specified target variable. After training, predictions were made for the outcome variable, and the R-squared value was calculated to quantify the variance explained by the model.

### Macronutrient multivariate regression model

In the analysis of macronutrient intake and its impact on incremental area under the curve for glucose, an Ordinary Least Squares (OLS) regression was conducted with carbohydrates, fats, and proteins as independent predictors and iAUC as the dependent variable. Prior to regression, a Variance Inflation Factor (VIF) analysis was conducted to ensure low multicollinearity among predictors, with all VIFs found to be less than 5, affirming the distinct contribution of each macronutrient to the model (Sheather, S. 2009. A modern approach to regression with R).

### Model robustness analysis

We were interested in the effect of the number of participants and the duration of tracking on model performances. We evaluated the performances on the model configuration consisting of only the glycemic and the dietary (temporal and compositional) data. The data were segmented by participant count, creating a gradient of training sets, each subsequently divided into different train-test fractions. We employed a gradient boosting algorithm to train models on these data subsets. The predictive accuracy of each model was quantified using the Pearson correlation coefficient. The correlation coefficients derived from this methodology were then visualized to illustrate the relationship between data set size and predictive accuracy, as shown in Supplementary Figure 2.

### SHAP analysis

To interpret the predictive model and assess the influence of individual features on the outcome, we employed SHapley Additive exPlanations (SHAP) using the *shap* package (version 0.42) in python (20). A TreeExplainer, specific to tree-based models such as XGBoost, was instantiated with our trained model to compute SHAP values for all features. These SHAP values represent the contribution of each feature to the model’s prediction for each observation, thereby quantifying the impact in a consistent and additive manner. Visualization of the distribution and impact of top 15 features was done using *summary_plot* function of *shap* package. Furthermore, dependence analysis was done for selected key features to observe the relationship between the magnitude of the feature and its SHAP value.

### Model robustness analysis

We conducted a series of experiments to understand the relationship between the size of the training set and the resulting model accuracies. The method entailed varying the proportion of the dataset through train-test splitting (from 10 to 90% of random samples of training data) and then subsequently examining the model performance. This process was repeated for a series of incrementally increasing participant counts, from 100 to the entire dataset, to simultaneously determine the effect of the number of participants on model performance.

## Results

### Glucose excursion profiles in relation to physiological features and in standardized meals

Glucose excursions are affected by many different factors stemming from nutritional, temporal and biological factors. Given that individual meal consumption varies in compositional proportions and portion sizes, conducting an initial investigation into standardized meals consumed at specific times of the day, where confounding factors are minimized, becomes a crucial preliminary step in examining PPGR. Hence, we investigated the glycemic impact of three standardized meals, i.e., (i) glucose drink, (ii) bread, and (iii) bread with butter to inspect each individual’s glucose response to standardized meals with similar carbohydrate content (approx. 50 g) but different fat and protein composition (Figure 1). The details of the standardized meals administration have been described in detail in our previous study (9), but briefly, participants consumed standardized breakfasts from days 2 to 7 of the first week, avoiding alterations and post-meal activities for 2 h. Depending on their assigned cohort, participants consumed either 6 or 12 standardized breakfasts and 2 or 4 glucose drinks, with some participants repeating the protocol in week 3 of their tracking phase.

**FIGURE 2:**
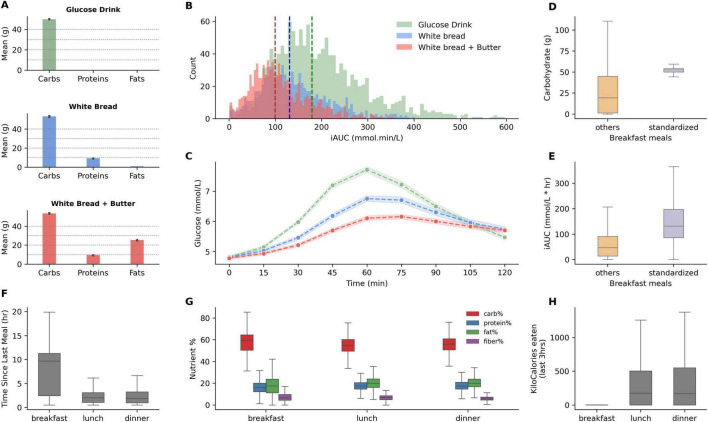
Macronutrient composition and glycemic response of standardized meals, and nutritional features comparison between breakfast, lunch, and dinner meals. **(A)** Displays the mean grams of carbohydrates, proteins, and fats in the standardized breakfasts: A glucose drink, white bread, and white bread with added butter. **(B)** Illustrates the distribution of postprandial incremental area under the curve (iAUC) for glucose after consumption of each standardized breakfast. Dashed vertical lines indicate the median iAUC for each type, demonstrating the shift in glucose response. **(C)** Displays glucose responses for the standardized meals (shaded regions highlight confidence intervals at 99%). **(D,E)** Compare carbohydrate and iAUC values for standardized breakfasts vs. other breakfast meals. **(F)** Presents the interquartile range for the time elapsed since the last meal for breakfast, lunch, and dinner. **(G)** Boxplots illustrate the proportion of macronutrients—carbohydrates, proteins, fats, and fiber—consumed during breakfast, lunch, and dinner. **(H)** Boxplot for past 3-h kcal consumption for breakfast, lunch, and dinner meals.

It is well known that carbohydrate content increases PPGR, while increasing fat proportions tend to reduce it (2). Figure 2A presents the macronutrient content in grams for these standardized meals, showing a consistent carbohydrate content across meals with variations in protein (approx. 10 g) and fat content (approx. 25 g) introduced by the addition of butter to white bread. Figure 2B depicts the distribution of the incremental area under the curve (iAUC) for glucose for standardized meals. As expected, median iAUC for glucose drinks (at 180 mmol⋅min/L) were highest, followed by white bread only and white bread with butter, with median iAUC at 130 and 99 mmol⋅min/L respectively, demonstrating that the combination of fat and carbohydrates may have modulatory behaviour on postprandial glucose excursions, while liquidized form of glucose exhibited higher PPGR. Correspondingly, the glucose response profiles of these meals (Figure 2C) also demonstrate that white bread with butter had an attenuated profile even when compared to simple white bread. On the opposite side of the spectrum, glucose drinks started to increase PPGR sharply within 30 min of consumption and had much higher peaks, and higher iAUC. Multiple regression analysis on dietary macronutrients and iAUC on the entire dataset demonstrated that each additional gram of carbohydrates increased iAUC by 1.08 mmol⋅min/L. In contrast, each additional gram of fat and protein decreased iAUC by 1.54 and 3.27 mmol⋅min/L, respectively, indicating inverse relationships with postprandial glucose levels, wherein the effect of protein was double.

We observe high inter-individual variability for each standardized meal, especially for glucose drinks, reflected by the high variance in iAUC (Figure 2B). Standardized meals have larger carbohydrate content than what most participants typically consumed for breakfast (Figure 2D), and lower iAUC was observed for regular breakfast meals (Figure 2E). Since breakfasts are normally not preceded by recent consumption (Figure 2F), the nutritional features of previous hours for such meals are absent (Figure 2H). Lunch and dinner, on the other hand, do not differ strongly when considering past meal characteristics and nutrient composition (Figure 2G).

### Combinatorial feature analysis

In order to best identify the relevant set of features for PPGR, a combinatorial approach was implemented and overall model performance was assessed for each combination of feature sets. Each feature set is derived from corresponding data sources such as glycemic metrics, dietary metrics, microbiome, sleep and physical activity datasets (see Material and methods section for feature generation details). Most model features are mainly comprised of features that were defined in Zeevi et al. (4), Berry et al. (5), Tily et al. (7), except for the ones related to microbiome, sleep and physical activity. Adding a set of features to a model typically led to a reduction in the number of observations, as only those with non-null values for these features were kept.

**FIGURE 3:**
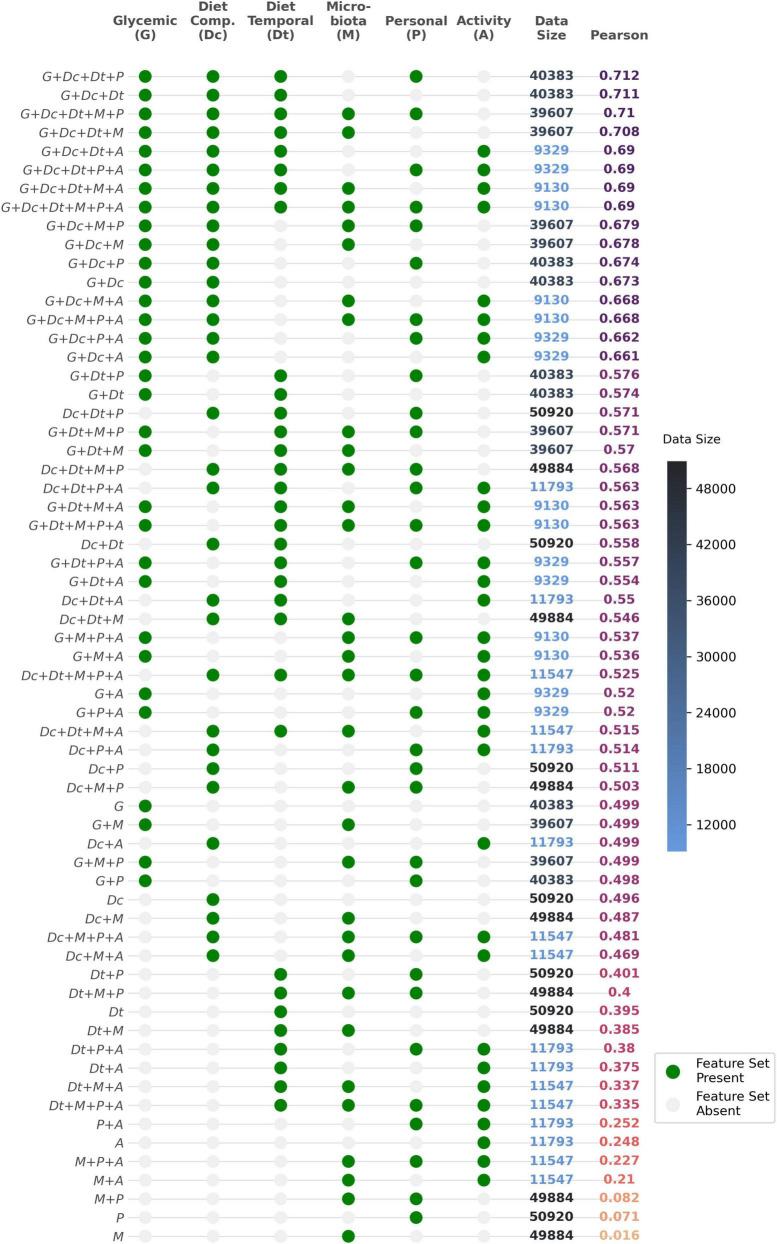
Different model implementations and corresponding dataset size and Pearson correlation scores. Presence of a particular feature set is shown in green. M represents microbiome features (which includes alpha diversity indices and 10 principle components of unweighted UniFrac distances). Dataset size refers to the number of observations, i.e., food loggings across all users, without missing data in any of the features. Feature list for each variable group are detailed in Supplementary Table 1.

In Figure 3, we can see that combinations that both contained glycemic (*G*), diet compositional (*Dc*) and diet temporal (*Dt*) feature sets had the best performances (> 0.68 Pearson correlation). Removing any one of those three feature sets results in lower performance. Even though the best performing combination also contains the personal feature set (i.e., demographic variables such as age, BMI, etc.) along with *G*, *Dc* and *Dt*, its relative performance increase is negligible. The second-best combination, lacking this feature set, is almost identically in performance. This is also true for the microbiome feature set, whose addition to either the *G* + *Dc* + *Dt* + *P* or *G* + *Dc* + *Dt* models provides no improvement to their performance. The observation that the addition of personal, microbiome and activity features does not increase model performance could mean that these feature sets are non-contributory; alternatively, other feature sets might already capture the effect of any contributory factors. On the other hand, the observation that the addition of personal, microbiome and activity features even slightly decreases model performance is simply due to their reduced data size, since we remove observations containing missing values for either of these feature sets. This is corroborated by the fact that if we replace missing values with median values for the feature to maintain data size, then the performances increase (data not shown).

**FIGURE 4:**
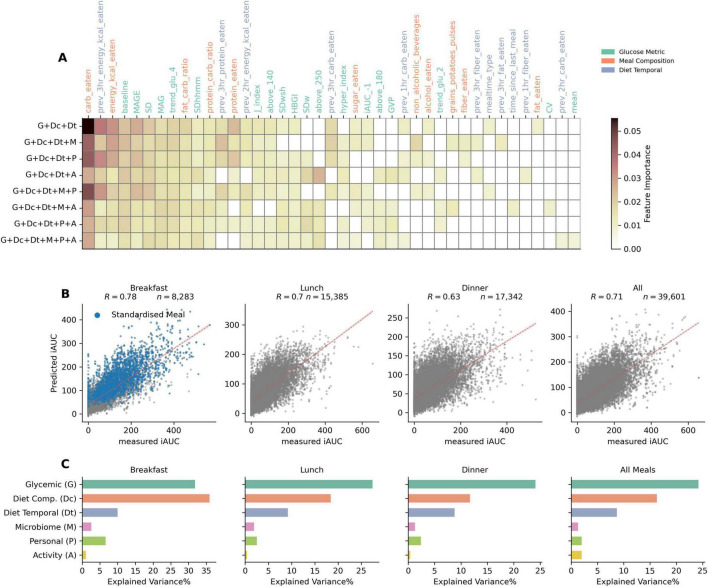
Feature importances for top features across different model combinations, correlation between predicted and measured PPGR for different meal timings (using gradient boosted tree models), and explained variance% (R^2^) of different feature sets (using linear regression models). **(A)** Heatmap of top features by their importance in different model combinations containing glycemic, compositional dietary, and temporal dietary features. **(B)** Scatter plots comparing the predicted iAUC against the measured iAUC for breakfast, lunch, dinner, and all meal logged data. Pearson correlation coefficient shown as “R” indicates the strength of the relationship. Blue dots in breakfast data demarcates standardized meals data points. The red dotted line represents the line of best fit, illustrating the predictive accuracy of the model for each meal timing type. **(C)** Explained variance (R^2^ in%) for different feature sets: Glycemic, Diet Composition, Diet Temporal, Microbiome, Personal (i.e., demographic such as age, BMI etc.) and Activity (sleep and physical), over meals consumed during different times of the day.

Taking only carbohydrates into the model gives a Pearson correlation score (R) of 0.46. Moreover, the model with only the two dietary feature sets, *Dc* and *Dt*, gives a *R* = 0.56. This highlights the overall importance of just using nutritional features in predicting iAUC. Models with just one of those feature sets yield *R* = 0.49 (*Dc*) and *R* = 0.39 (*Dt*). The glycemic feature set (*G*) alone provides a Pearson correlation score of 0.5, underlying the protective potential of past glucose values. Combining these three feature sets *G*, *Dc*, and *Dt*, yields the maximal model performance with a *R* = 0.71, higher than what was reported by other studies such as *R* = 0.68 by Zeevi et al. (4), *R* = 0.62 by Mendes-Soares et al. (6), and *R* = 0.64 by Tily et al. (7). Top feature importances for combinations containing *G*, *Dc* and *Dt* feature sets are shown in Figure 4A. The most important factors across all these model combinations are carbohydrate content, glucose baseline, previous 3 h energy kcal consumed, and past 4 h glucose trend.

### Model performances based on meal times

To further evaluate the performances based on temporal segregation of logged meals, i.e., split by breakfast, lunch and dinner, we trained and tested the model on each of these data splits. The *G* + *Dc* + *Dt* + *P* model was used to train and test on data segregated temporally. Scatter plots in Figure 4B show that the Pearson correlation between measured and predicted iAUC is *R* = 0.78 for breakfast, *R* = 0.7 for lunch, and *R* = 0.63 for dinner. While there appears to be a drop in performance over the day, it’s important to note that the correlation observed with regular, non-standardized breakfast alone is *R* = 0.67 (and *R* = 0.68 for standardized breakfast meals). Thus, when excluding standardized breakfasts, the model performance is quite comparable across meal times. The slight drop in performance for dinner may be attributed to the influence of accumulated confounding events affecting PPGR later during the day, such as physical activity or stress and fatigue, for which we have low amounts data, or none.

For each meal time, we examined the factors influencing PPGR through variance explained using regression analysis (Figure 4C). Relative to other feature sets, glycemic features exhibited the greatest explained variance for all types of meal times, with the exception of breakfast. In the case of breakfast, their explanatory power was similar to that of the diet composition feature set. For both glycemic and meal composition feature sets, a gradual decrease in explained variance was observed with increasing time of day. For the glycemic feature set (*G*), explained variance dropped from 31% for breakfast to 27% for lunch and < 25% for dinner. For the meal composition feature set (*Dc*), the explained variance dropped more sharply, from 33% for breakfast, to 15% for lunch, and 10% for dinner. The explained variance from the demographic/personal feature set was also higher in breakfast than in lunch and dinner. The activity feature set showed negligible effect in all cases.

### Comprehensive feature impact analysis on iAUC predictions

**FIGURE 5:**
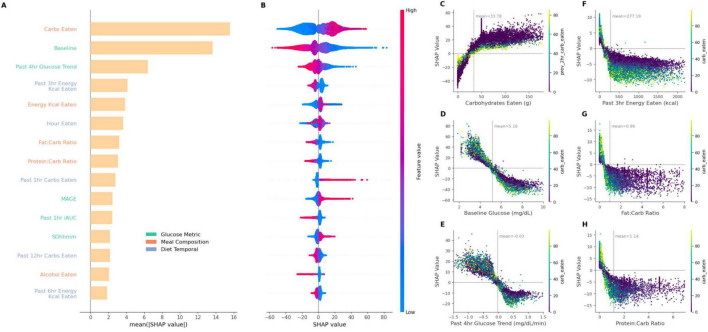
SHAP summary plot correlating feature impact on iAUC and SHAP dependency plots showcasing the relationship between six key features and their SHAP values. **(A)** provides the mean SHAP value, i.e., impact on predicted iAUC, for top 15 features in the XGBoost model of glycemic and dietary features sets implementation (*G* + *Dc* + *Dt* + *P*). **(B)** plot visualizes the directionality of SHAP values with the corresponding feature magnitude depicted by color intensity. **(C–H)** display the SHAP dependency plots of different features on iAUC prediction, highlighting the ranges of features which contribute toward prediction power [dots are additionally colored according to carbohydrates eaten, or previous 3 h carbohydrate eaten in the case of **(C)**]. (C) Carbohydrate intake demonstrates a significant increase in PPGR impact as carbohydrate consumption rises. The peak at around 50 g corresponds to the standardized breakfasts. **(D)** Baseline glucose and **(E)** glucose trend from 4 h prior show inverse influence on SHAP values. **(F)** Time since the last meal presents a decreasing pattern, suggesting its diminishing impact over time. **(G,H)** Show dependency plot for past 3 h energy kcal and protein-carb ratio. Each plot is annotated with a mean line for the corresponding features. Horizontal zero lines highlight the neutral point of no impact.

The identification of feature importance in predictive modeling is accomplished through the application of SHapley Additive exPlanations (SHAP), a technique for distinguishing the contributions of individual features to model outcomes (20). Such approach not only enhances model interpretability but also mitigates the ‘black box’ nature often associated with complex machine learning models. As depicted in Figure 5A, the SHAP summary plot offers a detailed visualization of the relative impact of the main contributing features on model performance. As before, this feature impact investigation was carried out in the *G* + *Dc* + *Dt* + *P* model. The top three impacting features are carbohydrates, glucose baseline, and past 4 h glucose trend. Even in other models, these features were always the most important.

Carbohydrates content was identified as the most influential predictor (Figure 5A). High carbohydrate intake was generally associated with increased iAUC predictions as indicated by high positive SHAP values (and vice versa), reflecting the positive correlation between carbohydrate consumption and PPGR (Figure 5B). Interestingly, the baseline glucose (i.e., glucose measurement at the time of corresponding meal logging) and the 4 h glucose trend prior to a meal features demonstrated inverse relationships with iAUC prediction. Higher values of baseline glucose and the 4 h glucose trend displayed negative SHAP values (Figures 5C,D), suggesting a negative contribution to the iAUC prediction. In other words, as the baseline glucose increases beyond a certain point, it is as expected associated with lower iAUC. Conversely, lower baseline glucose values (< 4 mmol/L) and lower past 4 h glucose trends (< –0.5) had higher SHAP values, suggesting that lower baseline glucose contributes to an increase in the predicted iAUC. A similar trend is also observed when considering nutritional features such as past 3 h energy kcal consumed, fat-carb ratio and protein-carb ratio (Figures 5E–G), wherein larger values are associated with lower iAUC but with a diminishing effect beyond a certain point where further increases in their value do not lead to more predictive power. For these ratios and past 3 h energy consumption, both higher relative protein/fat content and larger recent energy intake produces lower iAUC.

### Model robustness analysis

To understand model performance dependency on data availability, we used the model *G* + *Dc* + *Dt* and downsampled test set size and participant size (see Materials and methods). For each configuration defined by the number of participants and test set size, the model was trained and the Pearson correlation scores were plotted in Supplementary Figure 2. For future research, engaging fewer participants typically means lower resource requirements. Reducing the test size, which simulates collecting less data per participant, further conserves resources. It is therefore interesting to explore the minimum amount of data required to ensure model performance remains robust and reliable without overextending resources.

As expected, decreasing the number of participants or the size of the training set led to poorer model performance. However, model performance was quite stable to a reduction in participant numbers. For most test size reductions, substantial performance drops were only observed below 300 participants. With high levels of test size, even a participant count of 100 led to good model performance. Overall, this suggests that both the number of participants and the amount of data collected per participant could be reduced substantially to achieve good model results. For instance, model performances with the number of participants greater than 700 have their lowest model performance at approximately 0.65 for a training size of just 10% of the data, which is equivalent to the score reported by other studies (6, 7). However, more data generally leads to better model performance, as indicated by the superior model accuracy when using the full data set.

## Discussion

In this study based on over 1,000 participants of a digital cohort on personalized nutrition (Food & You), we showed that postprandial glucose responses (PPGR) can be accurately predicted using only glycemic and dietary information collected *in situ*. This finding is particularly relevant in the context of the global increase in metabolic diseases, as it suggests a scalable and non-invasive method for developing personalized dietary recommendations. The feasibility of this approach is enhanced by increasingly available technologies like glucose sensors and AI-assisted food tracking apps, such as MyFoodRepo, which in our study facilitated precise data collection with high user adherence (9), showcasing potential for long-term dietary monitoring and personalized interventions. Our work thus integrates into a series of previous studies (4–7, 8) by following their feature processing methodologies, but underscores the importance of high-resolution, temporal nutrition data on PPGR prediction.

The impact of meal composition on PPGR has already been well substantiated (4, 3, 21). Because participants collected diet data with an app in real time, we were able to investigate the importance of temporal diet data, which would be challenging with traditional diet tracking tools such as food frequency questionnaires. We found that temporal diet features are important for the best performing models, and consistently emerged as the top contributing features in the SHAP analysis (Figure 5). In addition, an intuitive yet interesting aspect of our study is the inclusion of previous days glycemic metrics. We are not aware of PPGR prediction studies that incorporate glycemic metrics from previous days, such as MAGE, SD, or MAG as features into the model. Such glycemic metrics can be derived from CGM data and serve as important measures for diabetes management and other clinical conditions (22).

The fact that the exclusion of microbiome features did not notably impact the model’s performance is a particularly intriguing finding. The microbiome’s role in metabolic processes and glucose regulation has been shown before (4, 23), and previous PPGR prediction studies have suggested that the microbiome is an important feature of high model accuracy. However, in the present study, we used an ablation approach to understand the relative importance of the various feature sets, and our results indicate that the available microbiome features—which also included PCA derived features from KEGG orthologs, pathways and enzyme information (from picrust)—were not necessary for achieving the best model performance. We attempted different methodologies to incorporate these microbiome features in the model, such as PCA-based features as used in (5), raw abundances, alpha diversities and most variable microbes, but their inclusion did not improve model performance. This may be because of the following reasons: (i) The Food & You study only collected 16S microbiota data, and their subsequent functional metagenomic features might not be sufficient to improve the model, whereas the addition of shotgun sequencing data (4, 8) or transcriptomics data (7) might; (ii) other feature sets, particularly those involving diet, might already capture the effect of any potential contributory microbiota factors; (iii) the microbiota as sampled in the Food & You study, i.e., from stool, is reflective of the microbiota in the sigmoid colon, whereas most glucose absorption happens in the duodenum and jejunum of the small intestine (24).

Similarly, incorporating sleep and physical activity features did not improve model performance, despite their recognized relevance in glycemic metabolic health (22, 25). In our study, when compared to food and glucose data, sleep and physical activity accounted for the lowest resolution of data as it was an optional aspect of the study. Consequently, incorporating this data led to a reduction in the overall training size per participant, which in turn, lowered model performance (see Figure 3, where, for example, the performance of model *G* + *Dc* + *Dt* + *A* was inferior to that of model *G* + *Dc* + *Dt*). However, when missing values in the activity features were imputed with their corresponding median values, the decline in performance was reversed. Generally, improving the collection of objective sleep and physical activity data could potentially enhance model performance (26).

The analysis of explained variance across meals, segmented by meal timings, offers insights into the factors influencing temporal PPGR. Glycemic features collectively explained most of the variance across all meal-timing types, at approximately 35%. Following this, meal composition explained an additional 20% of the variance for all recorded meals. This is in line with research that highlights the impact of macronutrient composition on glycemic outcomes (5). The SHAP analysis further underscored the influence of various factors, such as carbohydrate intake and baseline glucose levels, on iAUC predictions (Figure 5). Indeed, carbohydrate counting is a widely used method for diabetic management (27, 28), and our finding of a reasonable model performance of *R* = 0.46 with carbohydrate intake as the only feature demonstrates its efficacy as a first approximation. However, the SHAP analysis indicates that the predictive potential drops when carbohydrate content is low (< 25 g) (Figure 5A).

Carbohydrate counting is a straightforward method, but its predictive power for PPGR is relatively weak. The high performance of our carbohydrates-only model, compared to other studies, is likely due to Food & You participants tracking their food intake for at least 14 days, coupled with the high temporal resolution data collection through the MyFoodRepo app (9). The development of the *G* + *Dc* + *Dt* model, which requires only a glucose sensor and food tracking with the app, substantially improves model performance to *R* = 0.71. Given the ease of using CGMs for glucose sensing and the app for food tracking, we believe this approach offers a scalable solution for glucose management.

Our study has several limitations that warrant attention. It relied on data collected from a convenience sample in Switzerland (9), resulting in a lack of representativeness across several crucial dimensions. Future efforts should aim to broaden the diversity of the data. Additionally, the design of the cohort study protocol prioritized ease of use and high adherence, leading to decisions that compromised the data’s breadth and depth. For instance, the absence of blood sample collection restricted our ability to explore metabolic aspects in depth. Microbiota analysis was conducted at the 16S level due to resource constraints. The lack of standardization in physical activity tracking—specifically, not providing all participants with the same activity tracker—may have omitted vital indicators that could enhance model performance. Furthermore, this study was observational rather than interventional, meaning there is no experimental validation of our method’s effectiveness and performance yet, although such validation is in the planning stages.

Addressing these issues could potentially improve model performance further. Specifically, novel machine learning methods could further increase the predictive power of the models, a possibility we are actively exploring. However, there may be diminishing returns from incorporating an increasingly broad array of data sources to boost model performance. A trade-off between improving predictive power and the burden of collecting more individual data means that determining the optimal point becomes critically important. Although achieving ever-improving performance is appealing, data collection must remain cost-effective and as automated as possible to be scalable and widely adopted. Our model’s high performance, achieved with just two data sources—glycemia and diet, which comprises of not just carbohydrate content, but meal timing, previous meal consumption patterns, macronutrient ratios, and baseline glucose levels—all of which can be practically captured through a smartphone app and CGM to enable scalable, personalized nutritional recommendations.

## Data Availability

The authors acknowledge that the data presented in this article are not readily available because they contain personal information. Requests to access the datasets should be directed to the corresponding author at marcel.salathe@epfl.ch.

## Footnotes

1
https://www.diabetesatlas.org
