# Supplementary material for: Personalized glucose prediction using in situ data only
Source: Front Nutr. 2025 Jun 9;12:1539118. doi: 10.3389/fnut.2025.1539118 (PMC12183026; doi:10.3389/fnut.2025.1539118)
Supplement: Supplementary file 1 [file Data_Sheet_1.docx]

**Personalized glucose prediction using *in situ* data only**

*Rohan Singh^1^, Marouane Toumi^1^, Marcel Salathé^1^*

*^1^ Digital Epidemiology Lab, School of Life Sciences, School of Computer and Communication Sciences, EPFL, Switzerland*

Correspondence to: marcel.salathe@epfl.ch

# Supplementary Figures and Tables:


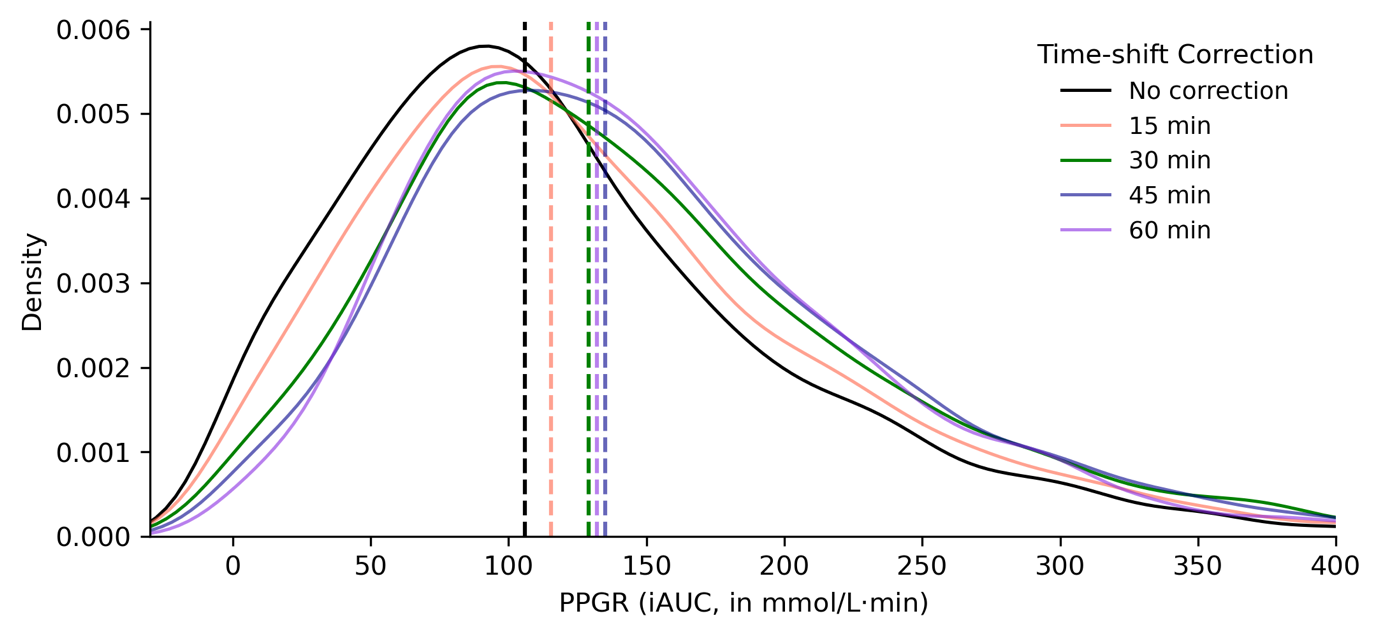


**Supplementary Figure 1: Kernel Density plot depicting the distribution of iAUC values for standardized meals computed using the different baseline search windows, e.g, 15 min into the past from logged meal time. Dashed vertical lines demarcate the median value for the corresponding iAUC time corrected distribution.**


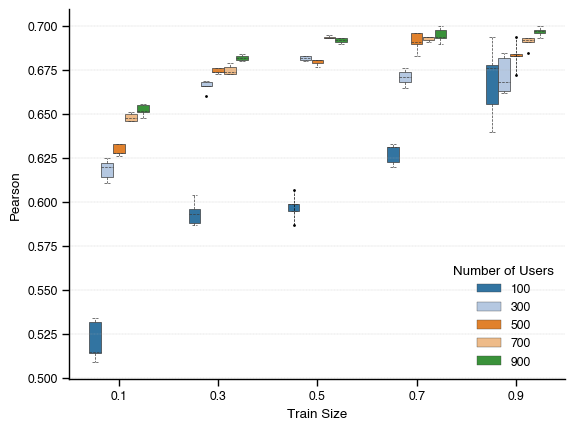


**Supplementary Figure 2: Boxplot depicting the relationship between the training set size and Pearson correlation coefficients for models trained with different numbers of participants.**

**Supplementary Table 1: Table describing the different features used in the study.**

| **Feature Name** | **Data type** |
| --- | --- |
| ADRR (Average Daily Risk Range) | Glucose |
| LBGI (Low Blood Glucose Index) | Glucose |
| HBGI (High Blood Glucose Index) | Glucose |
| hourly_auc (mean hourly AUC) | Glucose |
| COGI (COntinuous Glucose Monitoring Index) | Glucose |
| CONGA (Continuous Overall Net Glycemic Action) | Glucose |
| CV (Coefficient of Variation) | Glucose |
| CVmean (mean of daily coefficient of variation) | Glucose |
| CVsd (standard deviation of daily coefficient of variation) | Glucose |
| eA1C (estimated A1C) | Glucose |
| GMI (Glucose Management Indicator) | Glucose |
| GRADE (Glycemic Risk Assessment Diabetes Equation) | Glucose |
| GVP (Glucose Variability Percentage) | Glucose |
| hyper_index (Hyperglycemia Index) | Glucose |
| hypo_index (Hypoglycemia Index) | Glucose |
| IGC (Index of Glycemic Control) | Glucose |
| IQR (InterQuartile Range) | Glucose |
| J_index | Glucose |
| M_value | Glucose |
| MAD (Median Absolute Deviation) | Glucose |
| MAG (Mean Absolute Glucose) | Glucose |
| mean (mean of previous glucose values) | Glucose |
| MODD (Mean Of Daily Differences of blood glucose) | Glucose |
| above_140 (Percentage of values above 140 mg/dL) | Glucose |
| above_180 (Percentage of values above 180 mg/dL) | Glucose |
| above_250 (Percentage of values above 250 mg/dL) | Glucose |
| below_54 (Percentage of values below 54 mg/dL) | Glucose |
| below_70 (Percentage of values below 70 mg/dL) | Glucose |
| in_range_63_140 (Percentage of values between 63 mg/dL and 140 mg/dL) | Glucose |
| in_range_70_180 (Percentage of values between 70 mg/dL and 180 mg/dL) | Glucose |
| SD (Standard Deviation) | Glucose |
| SDw (Mean of daily Standard Deviation) | Glucose |
| SDhhmm (Standard Deviation of mean values at each time point across days) | Glucose |
| SDwsh (mean of Standard Deviation of each hour-long interval) | Glucose |
| SDdm (Standard Deviation of daily mean values) | Glucose |
| SDb (Standard Deviation between days, within timepoints ) | Glucose |
| SDbdm (Standard Deviation between days, within timepoints, corrected for changes in daily means) | Glucose |
| MAGE (Mean Amplitude of Glucose Excursions) | Glucose |
| trend_glu_1 (previous one-hour glucose slope) | Glucose |
| trend_glu_2 (previous two-hours glucose slope) | Glucose |
| trend_glu_4 (previous 4-hours glucose slope) | Glucose |
| iAUC_-1 (iAUC over the previous 1 hour) | Glucose |
| iAUC_-2 (iAUC over the previous 2 hours) | Glucose |
| iAUC_-4 (iAUC over the previous 4 hours) | Glucose |
| baseline (glucose value at the meal time) | Glucose |
| eaten_quantity_in_gram | DietComposition |
| energy_kcal_eaten | DietComposition |
| water | DietComposition |
| carb_eaten | DietComposition |
| fat_eaten | DietComposition |
| protein_eaten | DietComposition |
| fiber_eaten | DietComposition |
| alcohol_eaten | DietComposition |
| sugar_eaten | DietComposition |
| beta_carotene_eaten | DietComposition |
| calcium_eaten | DietComposition |
| cholesterol_eaten | DietComposition |
| fatty_acids_monounsaturated_eaten | DietComposition |
| fatty_acids_polyunsaturated_eaten | DietComposition |
| fatty_acids_saturated_eaten | DietComposition |
| folate_eaten | DietComposition |
| iron_eaten | DietComposition |
| magnesium_eaten | DietComposition |
| niacin_eaten | DietComposition |
| pantothenic_acid_eaten | DietComposition |
| phosphorus_eaten | DietComposition |
| potassium_eaten | DietComposition |
| salt_eaten | DietComposition |
| sodium_eaten | DietComposition |
| zinc_eaten | DietComposition |
| vitamin_b1_eaten | DietComposition |
| vitamin_b12_eaten | DietComposition |
| vitamin_b2_eaten | DietComposition |
| vitamin_b6_eaten | DietComposition |
| vitamin_c_eaten | DietComposition |
| vitamin_d_eaten | DietComposition |
| dairy_products_meat_fish_eggs_tofu | DietComposition |
| vegetables_fruits | DietComposition |
| sweets_salty_snacks_alcohol | DietComposition |
| non_alcoholic_beverages | DietComposition |
| grains_potatoes_pulses | DietComposition |
| oils_fats_nuts | DietComposition |
| fat_carb_ratio | DietComposition |
| protein_carb_ratio | DietComposition |
| mealtime_type | DietTemporal |
| time_since_last_meal | DietTemporal |
| hour_eaten | DietTemporal |
| prev_1hr_energy_kcal_eaten | DietTemporal |
| prev_1hr_carb_eaten | DietTemporal |
| prev_1hr_fat_eaten | DietTemporal |
| prev_1hr_protein_eaten | DietTemporal |
| prev_1hr_fiber_eaten | DietTemporal |
| prev_1hr_water | DietTemporal |
| prev_2hr_energy_kcal_eaten | DietTemporal |
| prev_2hr_carb_eaten | DietTemporal |
| prev_2hr_fat_eaten | DietTemporal |
| prev_2hr_protein_eaten | DietTemporal |
| prev_2hr_fiber_eaten | DietTemporal |
| prev_2hr_water | DietTemporal |
| prev_3hr_energy_kcal_eaten | DietTemporal |
| prev_3hr_carb_eaten | DietTemporal |
| prev_3hr_fat_eaten | DietTemporal |
| prev_3hr_protein_eaten | DietTemporal |
| prev_3hr_fiber_eaten | DietTemporal |
| prev_3hr_water | DietTemporal |
| prev_6hr_energy_kcal_eaten | DietTemporal |
| prev_6hr_carb_eaten | DietTemporal |
| prev_6hr_fat_eaten | DietTemporal |
| prev_6hr_protein_eaten | DietTemporal |
| prev_6hr_fiber_eaten | DietTemporal |
| prev_6hr_water | DietTemporal |
| prev_12hr_energy_kcal_eaten | DietTemporal |
| prev_12hr_carb_eaten | DietTemporal |
| prev_12hr_fat_eaten | DietTemporal |
| prev_12hr_protein_eaten | DietTemporal |
| prev_12hr_fiber_eaten | DietTemporal |
| prev_12hr_water | DietTemporal |
| faith_pd | Microbiome |
| shannon_entropy | Microbiome |
| pielou_evenness | Microbiome |
| observed_features | Microbiome |
| PC1 (Principle Component Beta Diversity) | Microbiome |
| PC2 | Microbiome |
| PC3 | Microbiome |
| PC4 | Microbiome |
| PC5 | Microbiome |
| PC6 | Microbiome |
| PC7 | Microbiome |
| PC8 | Microbiome |
| PC9 | Microbiome |
| PC10 | Microbiome |
| age | Demographic |
| age_group | Demographic |
| bmi | Demographic |
| bmr | Demographic |
| bmi_cat | Demographic |
| gender | Demographic |
| income | Demographic |
| cohabitants | Demographic |
| household_desc | Demographic |
| is_employed | Demographic |
| smoking | Demographic |
| health_state | Demographic |
| swiss_citizen | Demographic |
| education | Demographic |
| urbanity | Demographic |
| language | Demographic |
| folic_acid | Demographic |
| stress_level | Demographic |
| working_type | Demographic |
| screen_hours | Demographic |
| sleeping_problem | Demographic |
| general_hunger_level | Demographic |
| morning_hunger_level | Demographic |
| mid_hunger_level | Demographic |
| evening_hunger_level | Demographic |
| defecate_quantity_per_day | Demographic |
| has_eating_disorder | Demographic |
| antibiotics_treatment | Demographic |
| gut_disease | Demographic |
| sleep_time_diff_start | Activity |
| sleep_time_diff_stop | Activity |
| sleep_duration | Activity |
| pa_time_diff_start | Activity |
| pa_time_diff_end | Activity |
| pa_duration | Activity |

**Supplementary Table 2: Hyperparameters used for different model combinations.**

|  | **colsample bytree** | **eta** | **max depth** | **n_estimators** | **subsample** |
| --- | --- | --- | --- | --- | --- |
| gluc | 0.6 | 0.002 | 6 | 4000 | 0.7 |
| mealcomp | 0.6 | 0.001 | 6 | 4000 | 0.7 |
| mealtemporal | 0.6 | 0.002 | 6 | 4000 | 0.7 |
| microbiome | 0.6 | 0.001 | 6 | 1000 | 0.2 |
| demo | 0.6 | 0.001 | 7 | 1000 | 0.2 |
| activity | 0.6 | 0.001 | 6 | 1000 | 0.2 |
| gluc+mealcomp | 0.9 | 0.002 | 7 | 4000 | 0.5 |
| gluc+mealtemporal | 0.3 | 0.005 | 6 | 4000 | 0.9 |
| gluc+microbiome | 0.6 | 0.002 | 7 | 2000 | 0.9 |
| gluc+demo | 0.6 | 0.002 | 6 | 4000 | 0.5 |
| gluc+activity | 0.6 | 0.01 | 6 | 1000 | 0.5 |
| mealcomp+mealtemporal | 0.6 | 0.005 | 7 | 2000 | 0.7 |
| mealcomp+microbiome | 0.6 | 0.002 | 7 | 1000 | 0.2 |
| mealcomp+demo | 0.9 | 0.002 | 6 | 2000 | 0.2 |
| mealcomp+activity | 0.3 | 0.002 | 7 | 2000 | 0.7 |
| mealtemporal+microbiome | 0.6 | 0.001 | 7 | 4000 | 0.7 |
| mealtemporal+demo | 0.6 | 0.002 | 7 | 4000 | 0.5 |
| mealtemporal+activity | 0.6 | 0.001 | 7 | 2000 | 0.7 |
| microbiome+demo | 0.9 | 0.001 | 6 | 1000 | 0.9 |
| microbiome+activity | 0.6 | 0.001 | 7 | 4000 | 0.7 |
| demo+activity | 0.3 | 0.005 | 6 | 1000 | 0.2 |
| gluc+mealcomp+mealtemporal | 0.6 | 0.005 | 7 | 4000 | 0.7 |
| gluc+mealcomp+microbiome | 0.6 | 0.005 | 7 | 2000 | 0.7 |
| gluc+mealcomp+demo | 0.9 | 0.005 | 7 | 2000 | 0.5 |
| gluc+mealcomp+activity | 0.3 | 0.005 | 6 | 4000 | 0.7 |
| gluc+mealtemporal+microbiome | 0.6 | 0.005 | 7 | 2000 | 0.9 |
| gluc+mealtemporal+demo | 0.6 | 0.005 | 7 | 2000 | 0.7 |
| gluc+mealtemporal+activity | 0.3 | 0.01 | 6 | 1000 | 0.7 |
| gluc+microbiome+demo | 0.6 | 0.001 | 7 | 4000 | 0.7 |
| gluc+microbiome+activity | 0.3 | 0.005 | 6 | 1000 | 0.9 |
| gluc+demo+activity | 0.3 | 0.002 | 7 | 4000 | 0.7 |
| mealcomp+mealtemporal+microbiome | 0.9 | 0.002 | 7 | 4000 | 0.7 |
| mealcomp+mealtemporal+demo | 0.6 | 0.005 | 7 | 2000 | 0.7 |
| mealcomp+mealtemporal+activity | 0.3 | 0.002 | 6 | 4000 | 0.5 |
| mealcomp+microbiome+demo | 0.6 | 0.001 | 7 | 2000 | 0.2 |
| mealcomp+microbiome+activity | 0.3 | 0.001 | 7 | 4000 | 0.9 |
| mealcomp+demo+activity | 0.3 | 0.001 | 7 | 4000 | 0.7 |
| mealtemporal+microbiome+demo | 0.3 | 0.002 | 7 | 4000 | 0.9 |
| mealtemporal+microbiome+activity | 0.3 | 0.001 | 7 | 4000 | 0.9 |
| mealtemporal+demo+activity | 0.3 | 0.002 | 7 | 2000 | 0.7 |
| microbiome+demo+activity | 0.3 | 0.001 | 7 | 2000 | 0.7 |
| gluc+mealcomp+mealtemporal+microbiome | 0.6 | 0.005 | 7 | 4000 | 0.7 |
| gluc+mealcomp+mealtemporal+demo | 0.6 | 0.005 | 7 | 4000 | 0.7 |
| gluc+mealcomp+mealtemporal+activity | 0.6 | 0.005 | 7 | 4000 | 0.5 |
| gluc+mealcomp+microbiome+demo | 0.9 | 0.002 | 7 | 4000 | 0.5 |
| gluc+mealcomp+microbiome+activity | 0.3 | 0.002 | 6 | 4000 | 0.7 |
| gluc+mealcomp+demo+activity | 0.3 | 0.005 | 6 | 4000 | 0.9 |
| gluc+mealtemporal+microbiome+demo | 0.3 | 0.005 | 7 | 4000 | 0.9 |
| gluc+mealtemporal+microbiome+activity | 0.6 | 0.005 | 6 | 2000 | 0.7 |
| gluc+mealtemporal+demo+activity | 0.3 | 0.005 | 6 | 2000 | 0.7 |
| gluc+microbiome+demo+activity | 0.6 | 0.005 | 6 | 1000 | 0.7 |
| mealcomp+mealtemporal+microbiome+demo | 0.6 | 0.005 | 7 | 4000 | 0.7 |
| mealcomp+mealtemporal+microbiome+activity | 0.6 | 0.005 | 7 | 2000 | 0.7 |
| mealcomp+mealtemporal+demo+activity | 0.3 | 0.005 | 7 | 2000 | 0.9 |
| mealcomp+microbiome+demo+activity | 0.3 | 0.001 | 7 | 4000 | 0.7 |
| mealtemporal+microbiome+demo+activity | 0.3 | 0.001 | 7 | 4000 | 0.9 |
| gluc+mealcomp+mealtemporal+microbiome+demo | 0.6 | 0.005 | 7 | 4000 | 0.7 |
| gluc+mealcomp+mealtemporal+microbiome+activity | 0.3 | 0.005 | 7 | 4000 | 0.7 |
| gluc+mealcomp+mealtemporal+demo+activity | 0.3 | 0.005 | 7 | 4000 | 0.7 |
| gluc+mealcomp+microbiome+demo+activity | 0.3 | 0.005 | 6 | 2000 | 0.7 |
| gluc+mealtemporal+microbiome+demo+activity | 0.6 | 0.002 | 6 | 4000 | 0.7 |
| mealcomp+mealtemporal+microbiome+demo+activity | 0.3 | 0.002 | 7 | 4000 | 0.9 |
| gluc+mealcomp+mealtemporal+microbiome+demo+activity | 0.3 | 0.005 | 7 | 4000 | 0.7 |
